# NTNG1 Modulates Cisplatin Resistance in Epithelial Ovarian Cancer Cells via the GAS6/AXL/Akt Pathway

**DOI:** 10.3389/fcell.2021.652325

**Published:** 2021-07-01

**Authors:** Shanyu Fang, Yuanyuan Luo, Ying Zhang, Houmei Wang, Qianfen Liu, Xinya Li, Tinghe Yu

**Affiliations:** Laboratory of Obstetrics and Gynecology, The Second Affiliated Hospital, Chongqing Medical University, Chongqing, China

**Keywords:** NTNG1, cisplatin resistance, ovarian cancer, Axl, DNA repair

## Abstract

Cisplatin resistance is a challenge in the treatment of epithelial ovarian cancer. Here, clinical data showed that the level of netrin-G1 (NTNG1) in cisplatin-resistant cancer was higher than that in cisplatin-sensitive cancer (2.2-fold, *p* = 0.005); patients with a high NTNG1 level in cancer tissues had shorter progression-free survival (11.0 vs. 25.0 months, *p* = 0.010) and platinum-free interval (5.0 vs. 20.0 months, *p* = 0.021) compared with patients with a low level. Category- or stage-adjusted analyses demonstrated that the association between the NTNG1 level and prognosis occurred in type II or FIGO III/IV cancer. The basal level of NTNG1 in SKOV3/DDP cells (a cisplatin-resistant subline) was higher than that in SKOV3 cells; therefore, NTNG1 was overexpressed in SKOV3 cells, or silenced in SKOV3/DDP cells. Knocking in NTNG1 reduced the action of cisplatin to decrease cell death and apoptosis of SKOV3 cells, accompanied by upregulation of p-AXL, p-Akt and RAD51; however, opposite effects were observed in SKOV3/DDP cells after knocking down NTNG1. Co-immunoprecipitation demonstrated that NTNG1 bound GAS6/AXL. Silencing NTNG1 enhanced cisplatin effects *in vivo*, decreasing tumor volume/mass. These data suggested that a high NTNG1 level can result in cisplatin resistance in ovarian cancer cells via the GAS6/AXL/Akt pathway and that NTNG1 may be a useful target to overcome resistance.

## Introduction

Ovarian cancer is the most lethal gynecologic malignancy worldwide; epithelial cancer (EOC) accounts for >85% of cases. The standard treatment for EOC is cytoreductive surgery, followed by cisplatin (CDDP)-based chemotherapy. However, the 5-year survival rate is <40%, since the gradually increasing cisplatin resistance during treatment leads to treatment failure (Christie and Bowtell, 2017; Coburn et al., 2017).

Mechanisms of cisplatin resistance are only partially understood. Cisplatin commonly attacks DNA leading to apoptosis; therefore, an increase in DNA repair and activation of survival pathways can result in cisplatin resistance, and numerous candidate genes have been identified (Gasparri et al., 2018; Damia and Broggini, 2019). Understanding the functions of these molecules will help identify targets to overcome cisplatin resistance.

Netrin-G1 (NTNG1, also known as laminet-1) belongs to the family of netrins and interacts with diverse single-pass surface receptors to mediate cell repulsion, attraction, and adhesion (Sun et al., 2011). NTNG1 contains an extracellular N-terminal laminin-like domain and a C-terminal glycosylphos-phatidylinositol (GPI) anchor; NTNG1 predominantly tethers to the membrane through the GPI anchor, promoting outgrowth of thalamocortical axons (Yin et al., 2002; Lin et al., 2003). It has been shown that abnormal expression of the NTNG1 gene plays a role in the occurrence and recurrence of colorectal cancer, and that an alteration in NTNG1 activity is related to poor prognosis via disruption of the extracellular matrix (Yi et al., 2011; Sho et al., 2017). However, the role of NTNG1 in ovarian cancer remains unclear.

The receptor tyrosine kinase AXL triggers cancer progression. AXL interacts with its ligand growth arrest-specific 6 (GAS6), promoting cell adhesion, survival, and proliferation via activation of the ERK or Akt pathway (Graham et al., 2014). Recent data have indicated that AXL may participate in cisplatin resistance. AXL can prevent DNA damage due to drugs and promote DNA repair by upregulating the expression of RAD51, a key protein for homologous recombination (HR) (Balaji et al., 2017; Kim et al., 2017; Rose et al., 2020). High expression of AXL is associated with lower therapeutic responses and poorer prognosis in ovarian cancer; thus, AXL is a candidate molecule to conquer cisplatin resistance (Kim et al., 2015; Suh et al., 2015; Tian et al., 2021). However, underlying mechanisms are poorly understood.

Our protein interaction analysis showed that NTNG1 can interact with GAS6, suggesting that the role of NTNG1 may correlate with AXL. Here, the correlation between the expression level of NTNG1 and cisplatin response in ovarian cancer was evaluated using online datasets, and the role of NTNG1 in cisplatin resistance was explored with knock-in and knockdown experiments. Preliminary data indicated that NTNG1 bound GAS6/AXL to activate the Akt pathway, thereby modulating the response of ovarian cancer cells to cisplatin.

## Materials and Methods

### Bioinformatic Analyses

GSE45553 and GSE73935 datasets from the Gene Expression Omnibus (GEO) that contained mRNA profiles of cisplatin-sensitive and -resistant human ovarian cancer cell lines were analyzed. The GSE45553 dataset was for OVCAR-8 and OVCAR-8C, and GSE73935 was for A2780 and A2780-C; OVCAR-8C and A2780-C were cisplatin-resistant sublines. Interactions of the target gene and proteins were analyzed in the Biological General Repository for Interaction Datasets (BioGRID)^1^ (Oughtred et al., 2019).

The KM plotter^2^ was used to explore the relationship between the expression level of the target gene and progression-free survival (PFS) in patients with ovarian cancer (Zhou et al., 2019).

### Patients and Cancer Tissues

The use of human tissues was ethically approved by the local Institutional Review Board. Paraffin-embedded tumor tissues were collected from 67 EOC patients, who underwent cytoreductive surgery followed by cisplatin-based chemotherapy at the Second Affiliated Hospital, Chongqing Medical University (Chongqing, China) from August 2009 to June 2018. Clinical data (i.e., age, pathological type/grade, FIGO stage, therapeutic responses, and survival) were recorded. Resistance was defined as tumors that recurred or progressed within 6 months of the last dose, and sensitivity was defined as tumors that relapsed after 6 months (Matsuura et al., 2017). The therapeutic outcome was reflected using PFS and the platinum-free interval (PFI). PFS was the interval from the date of initial surgery to the date of progression/recurrence or last contact (censored), and PFI was the interval from the end of cisplatin treatment to the date of progression/recurrence or last contact (censored). PFS/PFI received stage- or category-adjusted analyses. Type I cancer included low-grade serous, clear cell, and endometrioid cancers; type II was high-grade serous cancer (Salazar et al., 2018).

### Detection of NTNG1 in Cancer Tissues With an Immunohistochemical Assay

An immunohistochemical assay was performed to detect NTNG1 in cancer tissues with a streptavidin–peroxidase kit (ZSGB-BIO, Beijing, China), using an anti-NTNG1 antibody (GeneTex, Irvine, CA, United States). The expression level of NTNG1 was quantified using the software Image-Pro Plus (Media Cybernetics, Rockville, MD, United States) and was expressed with the mean density (i.e., integrated absorbance/area). The cutoff value of a high/low expression level was determined using the receiver operator characteristic curve.

### Cells

Human EOC cell lines SKOV3 and SKOV3/DDP (identified by STR; Cell Bank, Type Culture Collect., Chin. Acad. Sci., Shanghai, China) were cultured in RPMI 1640 medium (Gibco, Beijing, China) enriched with 10% fetal bovine serum (Biol. Ind., Kibbutz Beit Haemek, Israel) at 37°C and 5% CO_2_. SKOV3/DDP was a resistant subline that can grow in the presence of 0.75 μg/mL of cisplatin (Yunnan Phytopharm., Kunming, China); cells were transferred to cisplatin-free medium for 5 days before performing experiments to avoid interference induced by residual drugs (Yu et al., 2015, 2016; Qian et al., 2019; Liu et al., 2020).

### Cell Viability

Cells were seeded in a 96-well plate (5.0 × 10^3^ cells per well) and then exposed to cisplatin (0, 0.5, 1.0, 2.0, 4.0, 8.0, and 16.0 μg/mL). Cell viability was determined with a CCK-8 assay (Dojindo Lab., Kumamoto, Japan) after 48 h. The half-maximal inhibition concentration (IC_50_) was calculated using the probit regression. For transfected cells, cells were subjected to cisplatin (IC_50_) and cell viability was determined after 24, 48, and 72 h.

### Cell Transfection

A lentiviral vector of shNTNG1 (GenePharma, Shanghai, China) was used to downregulate NTNG1 in SKOV3/DDP cells, and a lentiviral vector of NTNG1 (GenePharma) was adopted to upregulate NTNG1 in SKOV3 cells. shNTNG1, shNC, NTNG1, or NC was transferred into cells with the Polybrene kit (GenePharma). Puromycin (Solarbio Life Sci., Beijing, China) was added into the medium to remove uninfected cells, thereby obtaining stably transfected cells. The siRNA sequences were as follows: shNTNG1, 5′-CCAAGCCTCTCCAGGTTAA-3′, and shNC, 5′-TTCTCCGAACGTGTCACGT-3′. NC was the negative control (i.e., empty vector).

### Western Blotting

Proteins were extracted after cells were exposed to cisplatin (IC_50_) for 48 h using ice-cold RIPA buffer (Beyotime, Chongqing, China) supplemented with phenylmethanesulfonyl fluoride (PMSF); the concentration was determined with a BCA kit (Beyotime). Proteins were separated by SDS-PAGE and transferred to a PVDF membrane (Merck Millipore, Billerica, MA, United States). Primary antibodies were as follows: anti-NTNG1 (GeneTex), anti-RAD51 (Abcam, Cambridge, United Kingdom), anti-AXL/p-AXL (Cell Signaling Technology, Danvers, MA, United States), anti-Akt/p-Akt (Cell Signaling Technol.), anti-GAS6 (Bioss Biotechnology, Beijing, China), and anti-β-actin (Proteintech, Wuhan, China). The secondary antibody was a goat anti-rabbit IgG antibody (Abcam). Bands were analyzed with the software Image Lab (Bio-Rad Lab., Hercules, CA, United States). The density ratio was used to calibrate the level of a target protein, with β-actin as the reference.

To detect the expression level of NTNG1 after cisplatin exposure, proteins were extracted after SKOV3 or SKOV3/DDP cells were exposed to cisplatin (IC_50_ or 0.5 × IC_50_) for 48 h, or after SKOV3/DDP cells were cultured in cisplatin-free medium for 3, 5, 7, and 9 days.

### Cell Apoptosis

Cells were treated with cisplatin (IC_50_), and then apoptotic cells were detected using an Annexin V assay (Elabscience, Wuhan, China) after 48 h.

### Detection of γ-H2A.X Using an Immunofluorescent Assay

Cells were exposed to cisplatin (IC_50_) for 48 h, fixed with 4% paraformaldehyde for 30 min, blocked with 10% BSA for 1 h, and incubated with anti-γ-H2A.X antibody (Alexa Fluor-647 conjugate; Abcam) overnight at 4°C in the dark. Nuclei were counterstained with DAPI (Beyotime). Cells were observed under a confocal microscope (Nikon, Tokyo, Japan), and the fluorescence intensity was determined with Image-Pro Plus.

### Co-immunoprecipitation

Co-immunoprecipitation (coIP) was performed to validate the interaction between NTNG1 and GAS6/AXL. Protein A/G beads (MedChemExpress, Monmouth Junction, NJ, United States) were incubated with the primary antibody against NTNG1 (Santa Cruz Biotechnol., Dallas, TX, United States) with shaking for 1 h. NTNG1/NC-transfected SKOV3 cells were lysed in prechilled RIPA buffer supplemented with PMSF, protein A/G beads were added, and the mixture was shaken for 1 h. The beads were washed, and the eluted proteins were subjected to western blotting to detect NTNG1, GAS6, AXL, and p-AXL.

### *In vivo* Therapies

The use of laboratory animals was ethically and scientifically approved by the local Institutional Review Board in compliance with the Care and Use of Laboratory Animals. A total of 1.0 × 10^6^ NC− or NTNG1-transfected SKOV3 cells, and shNC− or shNTNG1-transfected SKOV3/DDP cells, were subcutaneously injected into the left armpit of 4-week-old female BALB/c nude mice (Cavens Lab. Anim., Changzhou, China), with five animals in each group. Cisplatin (10 mg/kg) was injected via a tail vein every 4 days at four times in groups NC + CDDP and NTNG1 + CDDP for SKOV3 tumors, and in groups shNC + CDDP and shNTNG1 + CDDP for SKOV3/DDP tumors; mice in the remaining groups received normal saline. The tumor volume was calibrated every 4 days [(length × width^2^)/2]. Animals were euthanized 4 days after the last dose; tumors were removed, weighed, and pathologically examined. NTNG1 and RAD51 proteins in tumor tissues were immunohistochemically detected.

### Statistics

Data were processed with the SPSS software (IBM, Armonk, NY, United States). Analysis of variance was used, and multiple comparisons were performed with the *t*-test. The correlation between the NTNG1 level and clinicopathological variables was analyzed with the chi-square test. PFS and PFI were evaluated with the Kaplan–Meier method. The difference was significant if the *p*-value was <0.05.

## Results

### A High Expression Level of NTNG1 in Cancer Tissues Indicated Chemoresistance and a Poorer Prognosis

Bioinformatic analyses of the GSE45553 and GSE73935 datasets indicated that NTNG1 was a candidate gene involved in cisplatin resistance in ovarian cancer; the BioGRID demonstrated an interaction between NTNG1 and GAS6. The expression level of NTNG1 in cisplatin-resistant cell lines was higher than in cisplatin-sensitive cell lines (log_2_ fold change, 2.3–4.0). The KM plotter indicated that a higher expression level of the NTNG1 gene was related to a shorter PFS of ovarian cancer patients in overall as well as in the subgroup that received cisplatin treatments (*p* = 0.005, *p* < 0.001) (Supplementary Figures 1, 2).

**FIGURE 1:**
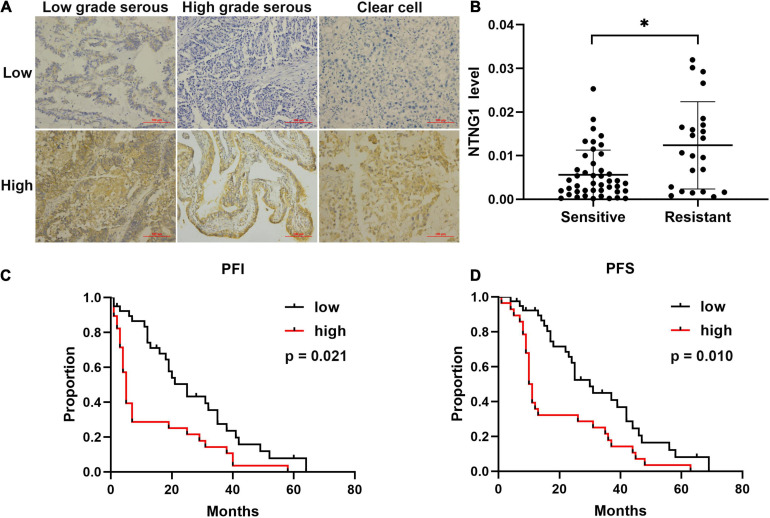
Level of correlated with cisplatin response and prognosis in patients with ovarian cancer (*n* = 67). **(A)** Representative immunohistochemical images of NTNG1 protein in cancer tissues; scale = 100 μm. **(B)** Expression level of NTNG1 in cisplatin-sensitive or -resistant cancer tissues; a higher level was observed in resistant cancers. **(C,D)** Kaplan–Meier analyses of platinum-free interval (PFI) and progression-free survival (PFS); patients with a high NTNG1 level in cancer tissues had shorter PFI and PFS compared with those with a low level. ^∗^*p* < 0.05.

To verify the aforementioned results, the correlation between the expression level of NTNG1 protein in cancer tissues and clinicopathological variables in 67 EOC cases was explored (Table 1). The NTNG1 level was higher in resistant cancers compared with sensitive cancers (0.0124 ± 0.0021 vs. 0.0056 ± 0.0009, *p* = 0.005) (Figures 1A,B); the cutoff value was 0.0066. Predictive values were 57.1% (95% confidence interval [CI]: 37.6–76.7%) and 82.1% (95% CI: 69.4–94.7%) when using a high level for resistance and a low level for sensitivity, respectively (*p* = 0.032). Patients with a high NTNG1 level in cancer tissues had shorter PFS [median: 11.0 (95% CI 8.9–13.0) vs. 25.0 (95% CI: 17.1–32.9) months, *p* = 0.010] and PFI [median: 5.0 (95% CI: 2.7–7.3) vs. 20.0 (95% CI: 13.9–26.1) months, *p* = 0.021], compared with those with a low level (Figures 1C,D). Adjusted analyses showed that the correlation between the high NTNG1 level and the poorer prognosis was observed in type II and FIGO III/IV cancers (Supplementary Figure 3). Overall, the data demonstrated that a high level of NTNG1 in cancer tissues indicated poorer therapeutic responses and outcomes.

**TABLE 1:** Clinicopathological characteristics and their associations with the expression level of NTNG1 in ovarian cancer tissues.

Clinicopathological variables	Case no.	NTNG1 expression level		*p*-value
		Low (*n* = 39)	High (*n* = 28)	
**Age (year)**				
<50	23	13 (56.5%)	10 (43.5%)	0.840
≥50	44	26 (59.1%)	18 (40.9%)	
**Histological type**				
Serous	59	34 (57.6%)	25 (42.4%)	0.386
Clear cell	7	5 (71.4%)	2 (28.6%)	
Endometrioid	1	0 (0.0%)	1 (100.0%)	
**Pathological grade**				
1/2	25	12 (48.0%)	13 (52.0%)	0.191
3	42	27 (64.3%)	15 (35.7%)	
**Category**				
Type I	32	17 (53.1%)	15 (46.9%)	0.420
Type II	35	22 (62.9%)	13 (37.1%)	
**FIGO stage**				
I/II	22	15 (68.2%)	7 (31.8%)	0.247
III/IV	45	24 (53.3%)	21 (46.7%)	
Cisplatin response*				
Resistant	23	7 (30.4%)	16 (69.6%)	0.001
Sensitive	44	32 (72.7%)	12 (27.3%)	

*Resistance was that tumors recurred or progressed within 6 months from the last dose, and sensitivity was that tumors relapsed after 6 months.

### A High NTNG1 Level Caused Cisplatin Resistance

**FIGURE 2:**
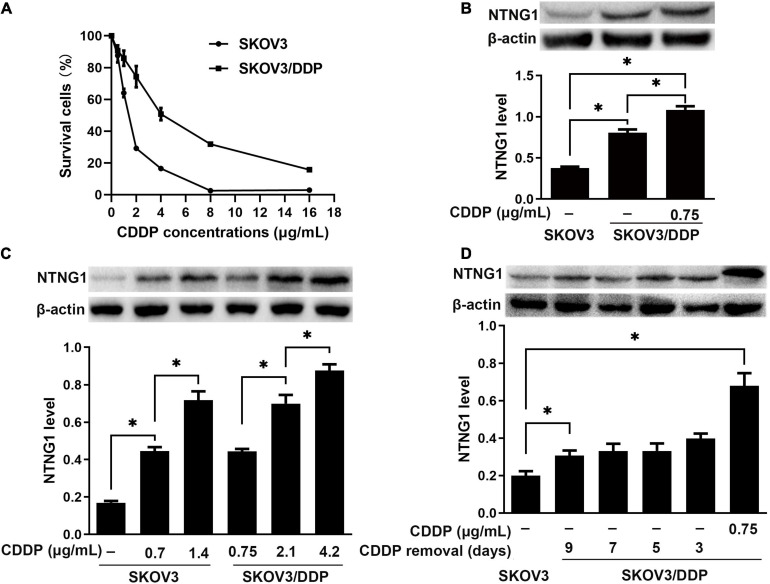
The level of NTNG1 was increased in cisplatin-resistant ovarian cancer cells (*n* = 3). **(A)** Cell survival (%) following cisplatin exposure; higher values were noted in SKOV3/DDP cells, confirming the resistance phenotype. **(B)** Expression level of NTNG1 was assayed by western blotting; the basal level in SKOV3/DDP cells (determined 5 days after cisplatin removal) was higher than that in SKOV3 cells; cisplatin induced its expression in SKOV3/DDP cells. **(C)** The level of NTNG1 increased with increasing concentration of cisplatin in SKOV3 and SKOV3/DDP cells. **(D)** The expression level of NTNG1 in SKOV3/DDP cells gradually decreased to the basal level following cisplatin removal. CDDP, cisplatin. ^∗^*p* < 0.05.

The IC_50_ values were 1.4 and 4.2 μg/mL for SKOV3 and SKOV3/DDP cells, respectively, confirming the resistance phenotype of SKOV3/DDP (Figure 2A). NTNG1 was detected in both cell lines, and the basal expression level in SKOV3/DDP was higher than that in SKOV3 (2.1-fold, *p* < 0.001) (Figure 2B). Therefore, SKOV3 and SKOV3/DDP were used for knock-in and knockdown experiments, respectively.

Following exposure to cisplatin, the level of NTNG1 dose-dependently increased in SKOV3 (2.6- to 4.2-fold, *p* < 0.001) and SKOV3/DDP (1.6- to 2.0-fold, *p* < 0.001) cells (Figure 2C). In SKOV3/DDP cells, this increased level gradually decreased to the basal level following the removal of cisplatin (*p* = 0.007) (Figure 2D).

**FIGURE 3:**
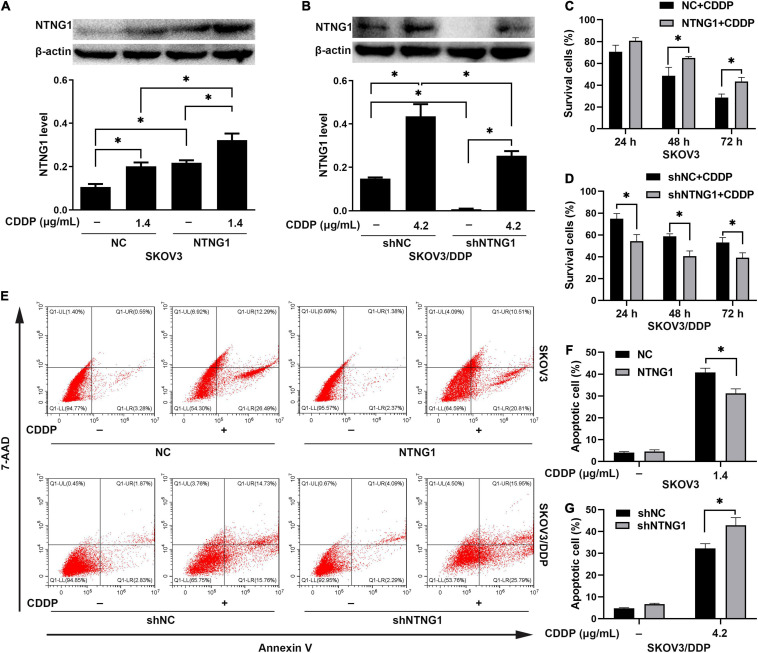
NTNG1 enhanced the action of cisplatin *in vitro* (*n* = 3). **(A,C)** The level of NTNG1 was increased in NTNG1-transfected SKOV3 cells; overexpression of NTNG1 increased the cell-survival percentage following cisplatin exposure. **(B,D)** The NTNG1 level was decreased in shNTNG1-transfected SKOV3/DDP cells; silencing NTNG1 decreased the cell-survival percentage following cisplatin treatment. **(E–G)** Apoptosis induced by cisplatin; the percentage of apoptotic SKOV3 cells decreased following overexpression of NTNG1, but increased in SKOV3/DDP cells after silencing NTNG1. CDDP, cisplatin. ^∗^*p* < 0.05.

Overexpression of NTNG1 decreased the percentages of dead and apoptotic cells induced by cisplatin in SKOV3 cells (*p* = 0.006–0.030, *p* = 0.004) (Figures 3A,C,E,F). These percentages were increased in SKOV3/DDP cells after silencing NTNG1 (*p* = 0.004–0.018, *p* = 0.011) (Figures 3B,D,E,G). Cisplatin-induced expression of NTNG1 was also observed following knock-in or knockdown. The findings demonstrated that NTNG1 was involved in cisplatin resistance.

### NTNG1 Promoted DNA Repair

**FIGURE 4:**
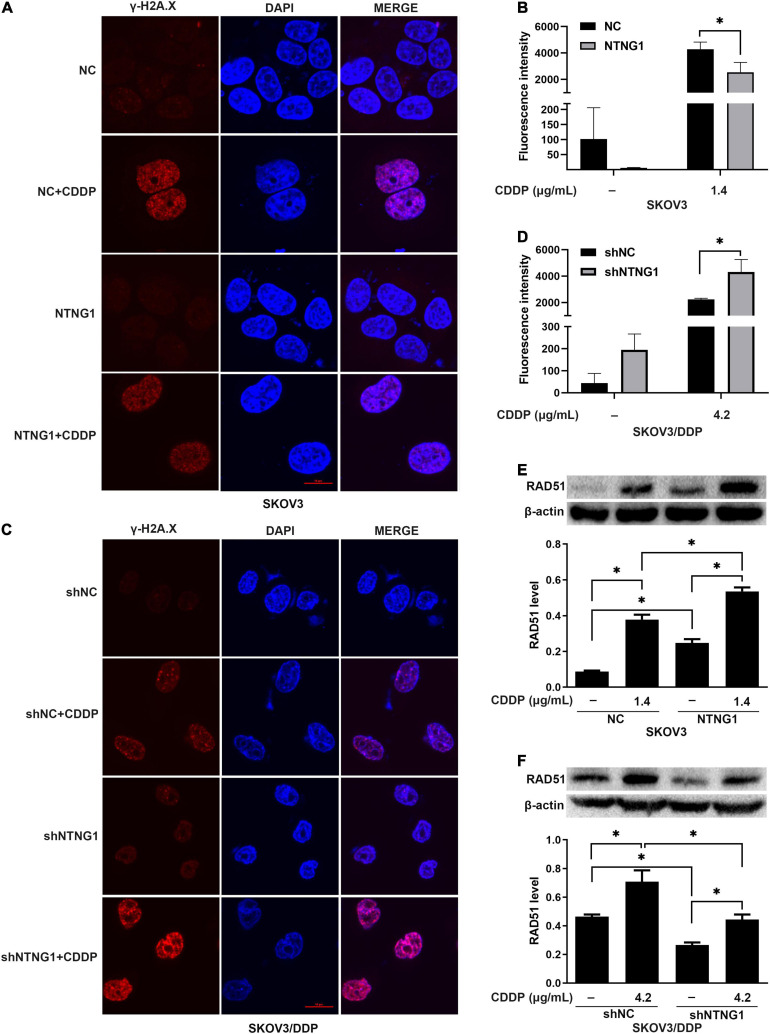
Effects of NTNG1 on DNA damage/repair (*n* = 3). **(A–D)** Immunofluorescent detection of γ-H2A.X; the level was increased in SKOV3 and SKOV3/DDP cells following cisplatin exposure; after cisplatin treatment, the level in NTNG1-transfected SKOV3 cells was lower than that in NC-transfected cells, but a higher level was observed in shNTNG1-transfected SKOV3/DDP cells compared with shNC-transfected cells; scale = 10 μm. **(E,F)** Cisplatin induced the expression of RAD51; following cisplatin exposure, a higher level was noted in NTNG1-transfected SKOV3 cells compared with NC-transfected cells, but a lower level was detected in shNTNG1-transfected SKOV3/DDP cells compared with shNC-transfected cells. CDDP, cisplatin. ^∗^*p* < 0.05.

DNA damage/repair was assayed by detecting γ-H2A.X and RAD51. γ-H2A.X was involved in the retention of repair complexes at sites of DNA damage, and RAD51 was a key molecule for HR (Bonner et al., 2008; Zhao et al., 2017). Cisplatin induced the formation of γ-H2A.X foci and an increase in the RAD51 level in both cell lines, i.e., initiating DNA repair. Overexpressing NTNG1 increased the RAD51 level in SKOV3 cells (*p* = 0.002), while the γ-H2A.X level decreased (*p* = 0.023) (Figures 4A,B,E). Silencing NTNG1 reduced the RAD51 level in SKOV3/DDP cells (*p* = 0.001), but the γ-H2A.X level was increased (*p* = 0.025) (Figures 4C,D,F). These data showed that NTNG1 upregulated the expression of RAD51, favoring DNA repair.

### NTNG1 Improved DNA Repair Through the AXL/Akt Pathway

**FIGURE 5:**
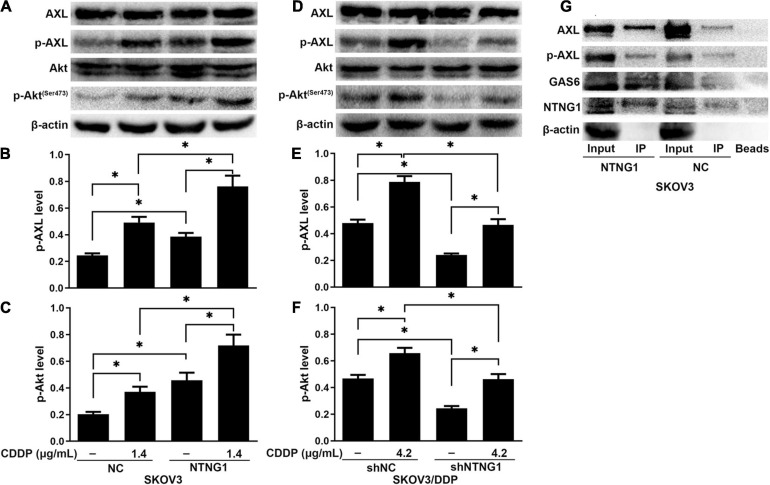
NTNG1 bound GAS6/AXL to activate the AXL/Akt pathway (*n* = 3). **(A–F)** AXL, p-AXL, Akt, and p-Akt were assayed by western blotting; the levels of p-AXL and p-Akt were increased following cisplatin exposure, indicating these molecules were involved in cell survival; such an inductive effect was enhanced in SKOV3 cells following overexpression of NTNG1 but was suppressed in SKOV3/DDP cells after silencing NTNG1. **(G)** Co-immunoprecipitation in SKOV3 cells; proteins were assayed by western blotting; the immunoprecipitate contained NTNG1, GAS6, and AXL/p-AXL; higher levels were noted following overexpression of NTNG1, confirming an interaction between NTNG1 and GAS6/AXL. CDDP, cisplatin. ^∗^*p* < 0.05.

The BioGRID database indicated an interaction between NTNG1 and GAS6, suggesting that NTNG1 can activate the AXL/Akt pathway to enhance DNA repair. Cisplatin caused DNA damage, inducing phosphorylation of AXL/Akt; the levels of p-Akt and p-AXL were increased in SKOV3 cells following overexpression of NTNG1 (*p* = 0.012, *p* = 0.013) (Figures 5A–C), but were decreased in SKOV3/DDP cells following silencing of NTNG1 (*p* = 0.001, *p* = 0.002) (Figures 5D–F).

To understand the mechanism of NTNG1 regulation of the AXL/Akt pathway, the interaction of NTNG1 and GAS6/AXL was validated by a coIP assay. The immunoprecipitate obtained from lysates of SKOV3 cells contained NTNG1, GAS6, and AXL/p-AXL; overexpression of NTNG1 increased the levels of GAS6 and AXL/p-AXL (Figure 5G). These data indicated that NTNG1 directly bound GAS6/AXL to activate the AXL/Akt pathway.

### NTNG1 Modulated the Action of DNA *in vivo*

**FIGURE 6:**
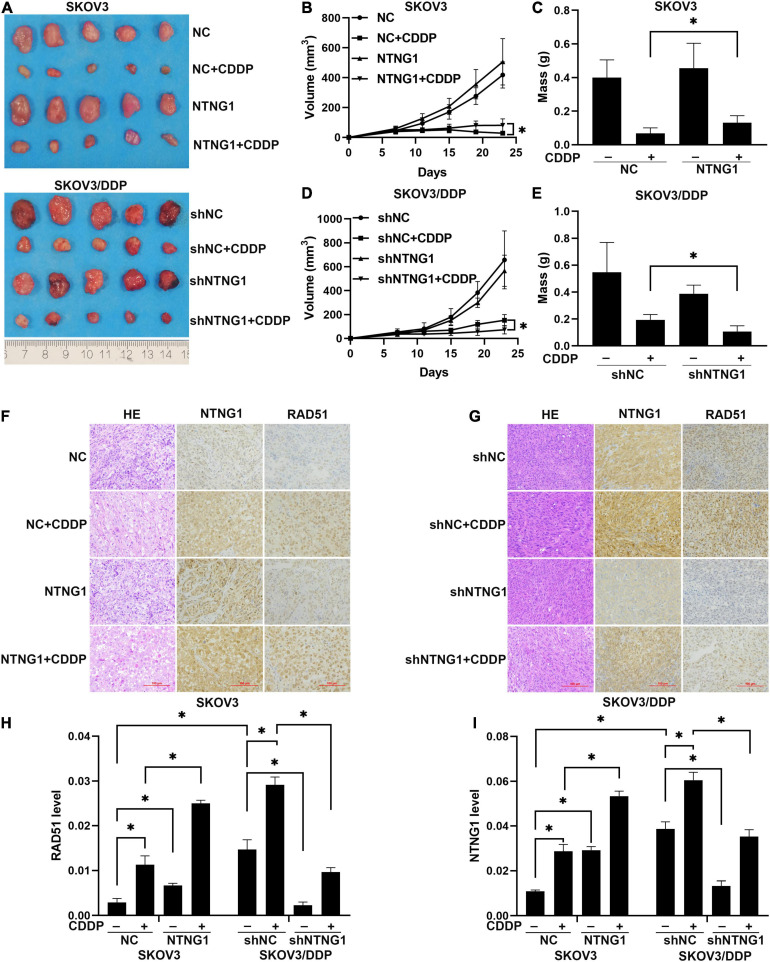
NTNG1 modulated the action of cisplatin in xenograft tumors (*n* = 5). **(A)** Image of SKOV3 and SKOV3/DDP tumors. **(B,C)** Volume and mass of SKOV3 tumors; values in group NTNG1 + CDDP were greater than those in group NC + CDDP, indicating a decrease in antitumor efficacy. **(D,E)** Volume and mass of SKOV3/DDP tumors; values in group shNTNG1 + CDDP were less than those in group shNC + CDDP, demonstrating a stronger anticancer action. **(F,G)** Immunohistochemical images of NTNG1 and RAD51 proteins in tumor tissues; scale = 100 μm. **(H,I)** Levels of NTNG1 and RAD51 proteins; cisplatin treatment induced the expression of RAD51; in SKOV3 tumors, levels of NTNG1 and RAD51 in group NTNG1 + CDDP were higher than those in group NC + CDDP; in SKOV3/DDP tumors, lower levels were detected in group shNTNG1 + CDDP compared with group shNC + CDDP. CDDP, cisplatin. ^∗^*p* < 0.05.

To determine the effect of NTNG1 on the action of cisplatin *in vivo*, NTNG1- or shNTNG1-transfected cells were injected into mice to form tumors. In SKOV3 tumors, overexpression of NTNG1 did not affect the tumor; tumor volume and mass in group NTNG1 + CDDP were greater than those in group NC + CDDP (*p* = 0.030, *p* = 0.029) (Figures 6A–C). In SKOV3/DDP tumors, silencing NTNG1 did not inhibit the tumor; smaller tumors were detected in group shNTNG1 + CDDP compared with group shNC + CDDP (*p* = 0.021, *p* = 0.009) (Figures 6A,D,E).

NTNG1 and RAD51 in tumor tissues were analyzed. These two proteins were present at a higher level in SKOV3/DDP tumors compared with SKOV3 tumors, and cisplatin treatment induced an increase in both tumor types. In SKOV3 tumors, levels of NTNG1 and RAD51 in group NTNG1 + CDDP were higher than those in group NC + CDDP (*p* < 0.001, *p* = 0.003) (Figures 6F,H,I); however, in SKOV3/DDP tumors, levels in group shNTNG1 + CDDP were lower than those in group shNC + CDDP (*p* < 0.001, *p* = 0.001) (Figures 6G–I). These data confirmed that the level of NTNG1 in tumor tissues determined the efficacy of cisplatin treatment.

## Discussion

Clinical data indicated that patients with a low NTNG1 level in cancer tissues had longer PFI and PFS and that cancers exhibiting a low NTNG1 level were sensitive to cisplatin. The NTNG1 level did not correlate with other clinicopathological variables. Thus, longer PFI and PFS resulted from a better therapeutic response. Category- or stage-adjusted analyses demonstrated that the correlation between a high NTNG1 level and poorer prognosis occurred only in type II or FIGO III/IV cancers. These two results were consistent. Here, type II cancer was high-grade serous cancer, which was frequently detected at stage III/IV (Garces et al., 2015). Type II cancer had gene mutations (e.g., TP53 and BRCA) and copy amplifications (e.g., MYC and CCNE1), which can cause cisplatin resistance (Brachova et al., 2013; Rojas et al., 2016; Singh et al., 2019; Gorski et al., 2020). The sample size was small, and therefore the present results should be validated in larger trials. Using a low level to show sensitivity had a higher predictive value compared with using a high level to indicate resistance, i.e., a low NTNG1 level can provide more information for clinical decisions.

Cisplatin attacked DNA to cause breaks, and double-strand breaks (DSBs) led to cell death via apoptosis; enhancing DSBs was a strategy to modulate cisplatin treatment and to overcome resistance (He et al., 2014; Wan et al., 2018; Qian et al., 2019). The γ-H2A.X foci formed at the DSB sites to favor an accumulation of repair molecules and were therefore used to monitor DSB repair (Liu et al., 2016). HR was the major pathway employed to repair DSBs induced by cisplatin; RAD51 was a key molecule in this pathway (Sugiyama and Kantake, 2009; Helleday, 2010; Lee et al., 2019). Levels of γ-H2A.X and RAD51 were upregulated following cisplatin exposure, i.e., cisplatin caused DSBs, initiating HR. Overexpression of NTNG1 increased the RAD51 level in SKOV3 cells, boosting HR; silencing NTNG1 decreased the RAD51 level in SKOV3/DDP cells, debasing HR; the expression pattern of NTNG1 determined the cells’ response to cisplatin. These results were consistent with alterations of the percentages of dead and apoptotic cells following knock-in or knockdown of NTNG1. The γ-H2A.X foci disappear after DSBs were repaired (Pintado-Berninches et al., 2019). Consequently, a lower level of γ-H2A.X was observed following overexpression of NTNG1, but a higher level was detected after silencing NTNG1. The present data showed that NTNG1 modulated sensitivity to cisplatin by adjusting HR capability.

Survival pathways were necessary for cell survival and may be involved in chemoresistance. The Akt pathway was such a pathway to prevent apoptosis (Zhang et al., 2016). Activation of Akt can induce the expression of RAD51 to enhance DNA repair, while inactivation of Akt downregulated RAD51 to augment the action of DNA-damaging drugs (Ko et al., 2016; Boichuk et al., 2020). AXL, highly expressed in multiple cancer types, can activate Akt to favor cell proliferation and chemoresistance (Li et al., 2014; Tian et al., 2016). AXL was the only known ligand of GAS6; binding of GAS6 to AXL activated the kinase domain of AXL, and downstream signaling pathways such as the Akt and ERK pathways were activated (Wang et al., 2016; Antony et al., 2018; Li et al., 2019). Activation of Akt and AXL was realized via phosphorylation. The BioGRID suggested that GAS6 be a target protein of NTNG1. This was supported by our coIP results, which demonstrated an interaction between NTNG1 and GAS6/AXL. Cisplatin induced an increase in the level of p-AXL and p-Akt, confirming their roles in cisplatin resistance of ovarian cancer cells; the inductive effect was amplified in SKOV3 cells following overexpression of NTNG1, and an opposite result was observed in SKOV3/DDP cells when silencing NTNG1. These findings suggested the following mechanism: NTNG1 interacted with GAS6/AXL, activating the Akt pathway, which upregulated the expression of RAD51 and improved the HR capacity, ultimately leading to cisplatin resistance.

*In vivo* data demonstrated that NTNG1 determined the therapeutic outcome of cisplatin: upregulation of NTNG1 decreased the therapeutic efficacy, but downregulation enhanced the anticancer action. These data were consistent with the results of *in vitro* therapies. The expression pattern of RAD51 protein displayed a similar trend. Thus, NTNG1 modulated the action of cisplatin by affecting HR. The therapeutic efficacy should be verified on an orthotopic ovarian cancer model to improve the clinical relevancy (Zhang et al., 2017; Liu et al., 2020). SKOV3/DDP represented acquired resistance, but resistance can be intrinsic in refractory ovarian cancer (Luvero et al., 2014; Cornelison et al., 2017). Thus, the role of NTNG1 in intrinsic cisplatin resistance should be explored.

Overall, the level of NTNG1 was higher in cisplatin-resistant ovarian cancer tissues compared with cisplatin-sensitive ones; patients with a high NTNG1 level in cancer tissues had shorter PFS and PFI. NTNG1 directly bound GAS6/AXL to regulate phosphorylation of AXL and Akt, upregulated the expression of RAD51, enhanced DSB repair, and eventually resulted in cisplatin resistance. Thus, NTNG1 was a target for ovarian cancer treatment, and inhibiting NTNG1 may be a useful strategy to overcome cisplatin resistance.

## Data Availability Statement

The original contributions presented in the study are included in the article/Supplementary Material, further inquiries can be directed to the corresponding author/s.

## Ethics Statement

The studies involving human participants were reviewed and approved by the Institutional Review Board of The Second Affiliated Hospital, Chongqing Medical University. Written informed consent for participation was not required for this study in accordance with the national legislation and the institutional requirements. The animal study was reviewed and approved by the Ethics Committee of Chongqing Medical University Approval.

## Author Contributions

SF designed the study and performed the experiments. YL, YZ, HW, and QL performed the experiments. SF and XL drafted the manuscript. TY designed the study and checked the manuscript. All authors have given approval to the final version of the manuscript.

## Conflict of Interest

The authors declare that the research was conducted in the absence of any commercial or financial relationships that could be construed as a potential conflict of interest.

## Footnotes

1 https://thebiogrid.org

2 http://kmplot.com/analysis/
