# Supplementary material for: NTNG1 Modulates Cisplatin Resistance in Epithelial Ovarian Cancer Cells via the GAS6/AXL/Akt Pathway
Source: Front Cell Dev Biol. 2021 Jul 1;9:652325. doi: 10.3389/fcell.2021.652325 (PMC8281315; doi:10.3389/fcell.2021.652325)
Supplement: Supplementary file 1 [file Image_1.pdf]

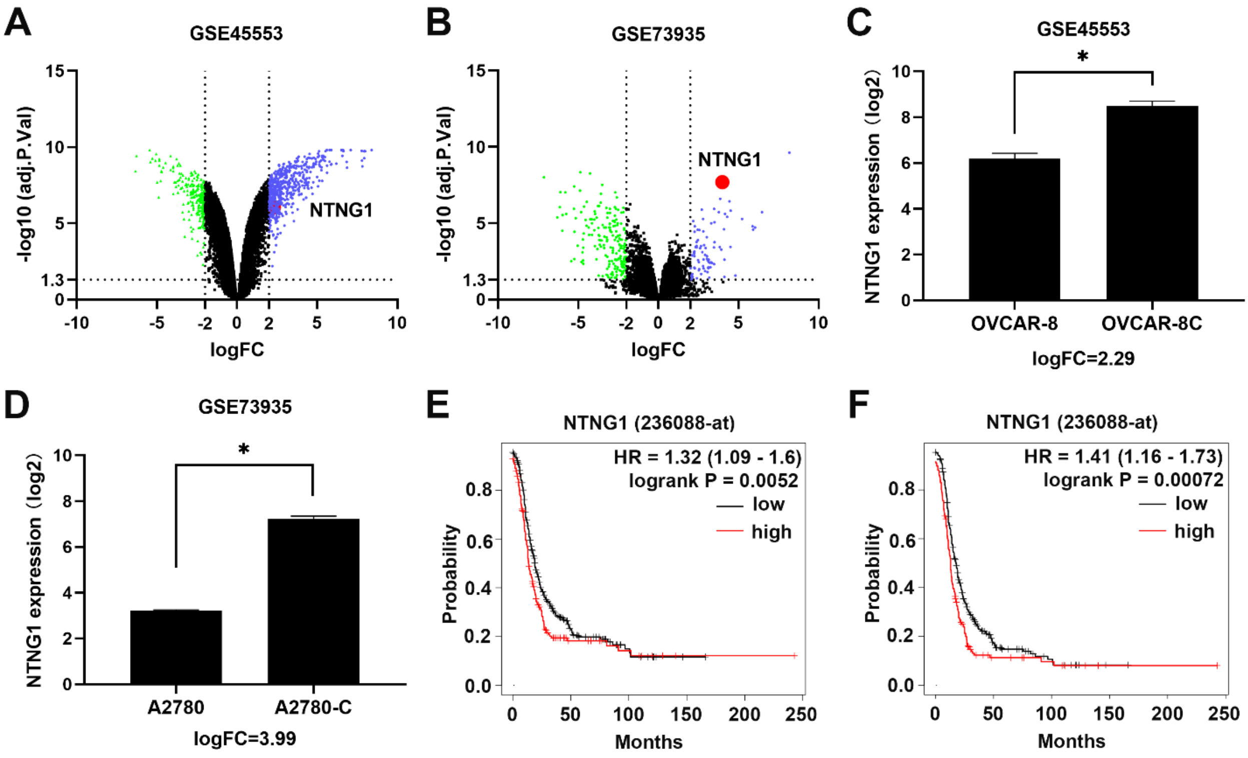

Figure 1 Bioinformatics analyses of the expression of NTNG gene in ovarian cancer. Volcano plots of gene expression profile in GSE45553 and GSE73935 datasets (***A, B***). The mRNA level of NTNG1 was higher in cisplatin-sensitive than that in cisplatin-resistant cells (***C, D***). Progression-free survival (PFS) in overall cases (***E***), and in the subgroup receiving cisplatin treatment (***F***): patients with a high NTNG1 level in cancer tissues had shorter PFS compared with those with a low level. *: $p < 0.05$.

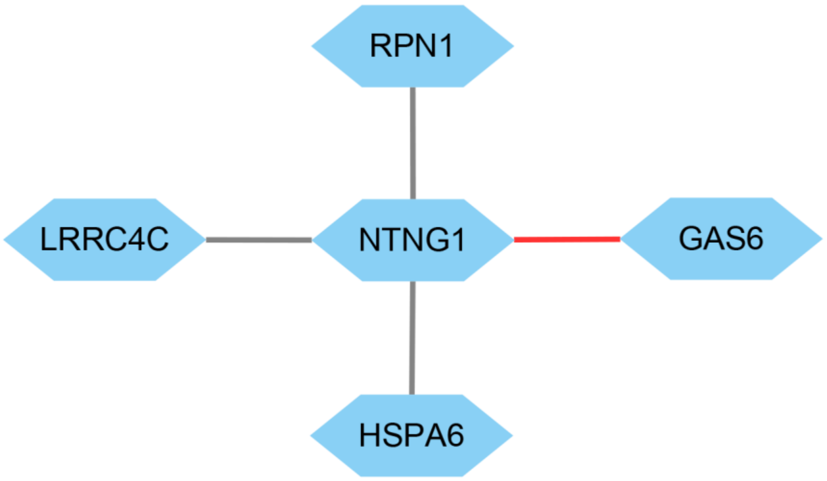

Figure 2 Proteins interacted with NTNG1 in the BioGRID database.

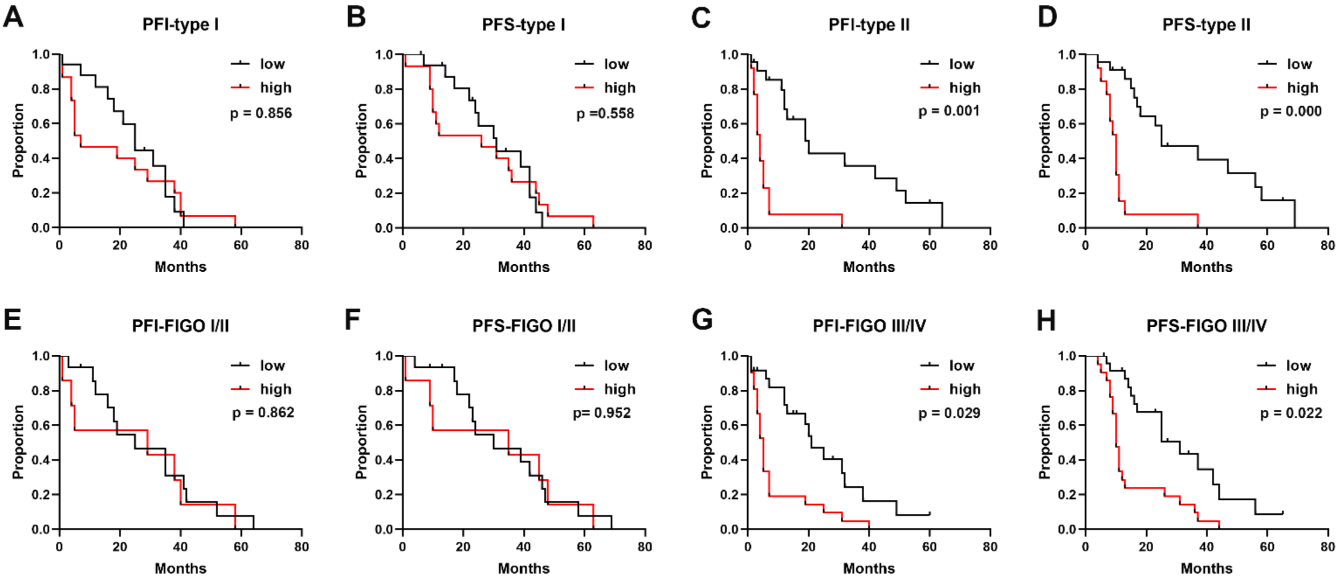

Figure 3 NTNG1 level correlated with prognosis in EOC patients. Platinum-free interval (PFI) and Progression-free survival (PFS) in type I (n = 32; low-grade serous: 24, clear cell: 7, endometrioid: 1) (***A, B***) and type II cancers (n = 35; high-grade serous) (***C, D***). PFI and PFS in FIGO I/II (n = 22) (***E, F***) and FIGO III/IV cancers (n = 45) (***G, H***): type II or FIGO III/IV cancer patients with a high NTNG1 level in cancer tissues had shorter PFI and PFS compared with those with a low level.
